# Detecting intracellular thiol redox state in leukaemia and heterogeneous immune cell populations: An optimised protocol for digital flow cytometers

**DOI:** 10.1016/j.mex.2018.10.013

**Published:** 2018-11-01

**Authors:** Alex J. Wadley, Rhys G. Morgan, Kate J. Heesom, Paul S. Hole, Steven J. Coles

**Affiliations:** a School of Science and the Environment, University of Worcester, Worcester, UK; b School of Sport, Exercise and Health Sciences, Loughborough University, Loughborough, UK; c School of Life Sciences, University of Sussex, John Maynard Smith Building, Brighton, UK; d Bristol Proteomics Facility, University of Bristol, Bristol, UK; e Institute of Cancer and Genetics, Cardiff University School of Medicine, Cardiff, UK

**Keywords:** Quantifying intracellular thiols by flow cytometry, Flow cytometry, Reactive oxygen species, Oxidative stress, Fluorescein-5 maleimide, Reductive stress

## Abstract

Flow cytometric methods for detecting and quantifying *reduced* intracellular thiol content using fluorescein-5-maleimide (F5M) in viable eukaryotic cells date back to 1983 (Durand and Olive [1]). There has been little development in these methodologies since that time, a period that has witnessed huge technological advances, particularly with the emergence of digital multi-parameter flow cytometric systems. Concurrent advancement in our understanding of redox regulation within eukaryotic cellular systems has also followed, whereby it is now accepted that cysteine thiols partake in redox reactions, which regulate protein activity and function (Groitl and Jakob (2014), Won et al. (2012)). Moreover, we are at the dawn of a new era in redox biology whereby the importance of ‘reductive stress’ in eukaryotic cellular systems is gathering momentum (Wadley et al. (2018) [4]). It is therefore critical that methods be continually advanced to better understand these concepts in more detail at the cellular level. Flow cytometry is a powerful technique that may be used for this purpose. Henceforth we have rejuvenated these methods to address modern scientific questions. In this paper, essential detail is provided on:

• The adaption of a protocol initially described by Durand and Olive [1] for use with modern digital flow cytometer configurations. Here we provide optimal conditions for labelling intracellular thiols with F5M for detection using digital flow cytometers. Our modifications avoid the use of methanol fixation thus preserving cell viability in single cell suspension cultures.
• Demonstration that flow cytometry can detect the gain and loss of *reduced* intracellular thiols in cells exposed to physiological doses of hydrogen peroxide mediated by glucose oxidase (Hole et al. (2013) [5]).
• Validation of F5M protein labelling by coupling method to confocal microscopy and downstream proteomics, thus permitting a powerful experimental platform for potential use with next generation flow cytometry e.g. CyTOF (Lin and Maecker (2018) [6]).

## Graphical abstract

**Figure:**
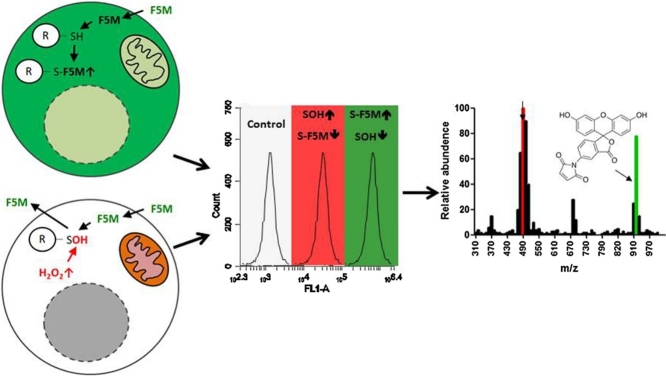

**Specification Table**

**Table:** 

Subject area	• *Biochemistry, Genetics and Molecular Biology*
More specific subject area	*Redox Biology, Cysteine Thiol Chemistry, Flow Cytometry and Mass Spectrometry*
Method name	*Original customized method name: Flow cytometry techniques for studying cellular thiols.*
Name and reference of original method	*If applicable, include full bibliographic details of the main reference(s) describing the original method from which the new method was derived. See the following citation* [1]
Resource availability	*n/a*

## Methods details

### Materials

1 Complete IMDM tissue culture medium: containing 10% (v/v) foetal calf serum (FCS), 2 mM l-glutamine, 100 U/ml penicillin and 100 mg/ml streptomycin (Sigma-Aldrich, Dorset, UK).
2 Hank’s balanced salt solution (Sigma-Aldrich, Dorset, UK; catalogue #H9269).
3 Dulbecco’s phosphate buffered saline (D-PBS) endotoxin tested, pH 7.0 (Sigma-Aldrich, Dorset, UK; catalogue #D8537).
4 Flow cytometry stain buffer (FSB): d-PBS adjusted to contain 2% (v/v) FCS, 0.1% (w/v) sodium azide and 5 mM EDTA (stored at 4 °C).
5 Ficoll-Paque PLUS (GE, Buckinghamshire, UK; catalogue #17144003) or Hisopaque 1077 (Sigma-Aldrich, Dorset, UK; catalogue #10771).
6 Trypan blue solution 0.4% (Sigma-Aldrich, Dorset, UK; catalogue #T8154).
7 Glucose oxidase from *Aspergillus niger* (Sigma-Aldrich, Dorset, UK; catalogue #G2133): 100,000 U/g.
8 *N-*ethylmaleimide (NEM): 1 mM stock in d-PBS stored at 4 °C protected from light (Sigma-Aldrich, Dorset, UK; catalogue #E3876).
9 Fluorescein-5 maleimide (F5M): 1 mM stock in d-PBS stored at 4 °C protected from light (ThermoFisher, Hampshire, UK; catalogue #62245).
10 Anti-human CD3-APC, clone SK7 (BioLegend, London, UK; catalogue #344812).
11 Multi-parameter digital flow cytometer.
  *Note*: in our laboratory we use an Accuri-C6 (BD-Accuri, Berkshire, UK); however, the method is compatible with other digital systems e.g. Guava^®^ EasyCyte (MerkMillipore, Hertfordshire, UK)
12 NuPAGE 4–12% SDS-PAGE gel, MOPS running buffer and SDS-PAGE LDS sample loading buffer (ThermoFisher, Hampshire, UK)

### Cells

1 Jurkat (ATCC^®^TIB-152^™^), human acute T-cell lymphoblastic leukaemia.
2 Donor human peripheral blood mononuclear cells (PBMCs).
  *Note*: full ethical approval and donor consent is required for the isolation of PBMCs from human blood, in strict accordance with the declaration of Helsinki [7].

### Experimental procedures

#### Analysing the effect of glucose oxidase mediated H_2_O_2_ on intracellular thiol oxidation using flow cytometry

The protocol described is based on a previous method described in Durand and Olive [1], which incorporated various fluorescent stains for the detection of *reduced* intracellular thiols, including fluorescein-5 maleimide (F5M). The current method is optimised for bench-top digital flow cytometers and allows low level detection of F5M in viable cells, which proved challenging using the previous method. The optimisation of the technique described herein is of particular importance, since some reactive oxygen species (ROS) e.g. hydrogen peroxide (H_2_O_2_) have the propensity to oxidise proteins with exposed cysteine residues, in turn altering their activity [8]. In the experimental model proposed, exposure of cells to physiological doses of exogenous H_2_O_2_ can be modelled in vitro by adding glucose oxidase (GOX) to the culture medium as previously described [5]. The addition of GOX in this way will metabolise glucose in the culture medium, generating H_2_O_2_ as defined by the following reaction [9]:

β-d-glucose + oxygen → d-glucono-1,5,-lactone + hydrogen peroxide

**Fig. 1:**
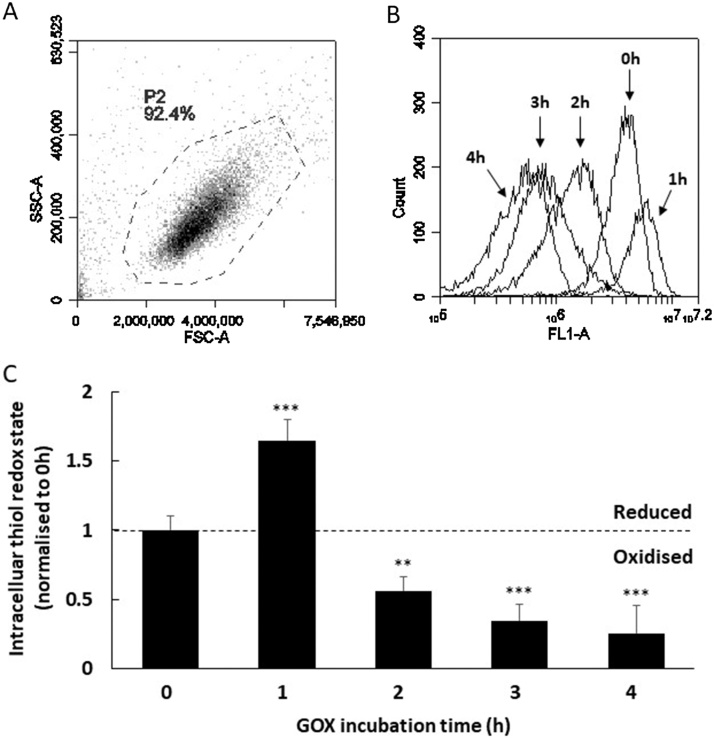
**The effect of exogenous GOX-generated H_2_O_2_ on the loss of intracellular reduced thiol using an in vitro immortalised Jurkat T-cell model**. (A) Representative FSC vs SSC flow cytometric profile of GOX treated Jurkat cells (P2). (B) Histogram (gated on P2) illustrates loss of F5M signal in Jurkat cells following incubation with 5 mU/ml GOX for 0–4 hour (h). Samples were taken for flow cytometric analysis after the following times; 0 h, 1 h, 2 h, 3 h and 4 h. Data were compared with 0 h to normalise the loss of F5M signal (see Table 1). (C) Chart illustrates normalised FL1 (laser: λ488 nm, filter: λ530/30 nm) MFI. An increase in F5M signal is noted at 1 h (suggesting a reductive spike), followed by progressive decrease in signal at 2 h–4 h (suggesting a loss of reduced thiols). Data shown are mean +/− SD of the mean (n = 3). Data analysed by Kruskal–Wallis one-way analysis of variance with Dunn’s post-hoc test, *p < 0.05, **p < 0.01 and ***p < 0.001.
19 Normalise the FL1 mean fluorescent intensity (MFI) values to ‘0 h’ in order interpret the flow cytometry data as shown in Fig. 1C and in Table 1.

Hydrogen peroxide is membrane permeable and can enter the cell mediating the oxidation of intracellular cysteine residues [10]. To demonstrate this, the example included uses the Jurkat immortalised T-cell line to monitor cellular cysteine thiol redox state over time in response to GOX generated H_2_O_2_. The method details are as follows:

1 Maintain Jurkat cells in 15 ml complete IMDM, at a density between 2 × 10^5^ and 10^6^ cells/ml at 37 °C and 5% CO_2_.
  Note: IMDM is particularly suited to this because of the high glucose concentration (4.5 g/l), which is a substrate for GOX.
2 Prepare a 10 mU/ml GOX solution and a 10 mg/ml catalase solution and store at 4 °C until required.
  Note: The catalase is used to confirm that H_2_O_2_ is mediating changes in thiol redox state. To prepare glucose oxidase solutions for in vitro H_2_O_2_ generation, it is recommended to obtain high purity glucose oxidase solid (e.g. Sigma cat no. 49180 or similar quality). Cheaper preparations can contain confounding contaminants such as catalase, which metabolises H_2_O_2_. In a Class 2 biological safety cabinet, dissolve solid GOX in tissue culture-grade water to a concentration of 1 U/ml (U = enzyme units). Sterilise by filtration and aliquot for storage at −20 °C and avoid freeze thaw. Prepare 10 mU/ml working solutions by thawing 1 U/ml stock aliquots at 37 °C for 1 min and diluting with tissue culture-grade water immediately prior to use. Discard excess. Note that H_2_O_2_ production generated by GOX in vitro can be measured and titrated to physiological levels using the H_2_O_2_-specific fluorescent probe Amplex UltraRed (Life Technologies, Hampshire, UK) [5].
3 Transfer the contents of a 24–48 h Jurkat cell culture flask (60–80% confluence) to a 15 ml tube and centrifuge at 100–300 × *g* for 10 min at room temperature in a bench-top centrifuge with swing-out rotor.
  Note: Jurkat cells are non-adherent and do not require Trypsin-EDTA treatment to dislodge from the flask surface.
4 Remove the supernatant and carefully suspend the pellet in 1 ml complete IMDM pre-warmed to 37 °C.
5 Perform a viable cell count using trypan blue and dilute the Jurkat cells to a viable density of 10^7^ cells/ml.
6 Add 100 μl of the diluted Jurkat cell suspension to five wells in a 96-well tissue culture treated plate and label ‘0 h’, ‘1 h’, ‘2 h’, ‘3 h’, ‘4 h’.
7 Add 98 μl to two wells in the same plate and label; ‘0 h +C’ and ‘4 h +C’. Note the “+C” refers to the addition of catalase. Thus there are 7 assay wells in total.
8 Add 2 μl of the 10 mg/ml catalase stock solution (prepared in step 2) to wells labelled ‘0 h +C’ and ‘4 h +C’ only.
9 To start the assay add 100 μl of the GOX solution (prepared in step 2) to wells labelled ‘4 h’ and ‘4 h +C’ and incubate at 37 °C and 5% CO_2_.
  Note: that this assay has been optimised to use IMDM because of the high glucose content (4.5 g/l), rather than RPMI-1640 which has much lower glucose content (2 g/l). This is important since GOX requires a glucose substrate to generate H_2_O_2_.
10 After 1 h, add 100 μl of the GOX solution to the ‘3 h’ well and return to the incubator.
11 Repeat after a further 1 and 2 h for wells labelled ‘2 h’ and ‘1 h’ respectively.
12 Finally, 1 h after the addition of GOX to ‘1 h’, add 100 μl of the GOX solution to wells labelled ‘0 h’ and ‘0 h + C’ and immediately transfer the contents of all 7 assay wells to a fresh 1.5 ml microcentrifuge tube. Centrifuge all at 100–300 × *g* for 5 min at room temperature.
13 Carefully remove supernatants and suspend the pellets in 1 ml d-PBS. Centrifuge all tubes at 100–300 × *g* for 5 min at room temperature.
14 Remove supernatants and suspend pellets in 1 ml pre-chilled 0.1 μM F5M solution and incubate all tubes for 20 min, on ice and protected from light.
15 Centrifuge tubes at 100–300 × *g* for 5 min at 4 °C.
16 Remove supernatants and suspend pellets in 1 ml pre-chilled FSB and centrifuge at 100–300 × *g* for 5 min at 4 °C.
17 Remove supernatants and suspend pellets in 100 μl FSB and go to flow cytometer. Keep tubes on ice and protect from light.
18 At the flow cytometer, acquire the ‘0 h’ tube first, followed by the others in chronological order. Draw a region around the Jurkat cell population as seen in Fig. 1A and create an FL1 (λ488 nm; λ530/30 nm) histogram gated on the region as seen in Fig. 1B. Non-viable cells <8% can be excluded from the flow cytometric analysis by the addition of a cell viability stain e.g. 7AAD.

**Table 1:** Raw flow cytometry data and normalised thiol redox state.

GOX incubation time (h)	Mean fluorescence intensity (MFI) in FL1 (arbitrary units)	Normalised intracellular thiol redox state (later time/0 h)^#^
0	3,163,900.56	0 h MFI/0 h MFI = **1**
1	5,409,960.52	1 h MFI/0 h MFI = **1.64** (p < 0.001)
2	1,777,116.26	2 h MFI/0 h MFI = **0.56** (p < 0.01)
3	1,102,371.66	3 h MFI/0 h MFI = **0.34** (p < 0.001)
4	807,302.74	4 h MFI/0 h MFI = **0.25** (p < 0.001)

**Fig. 2:**
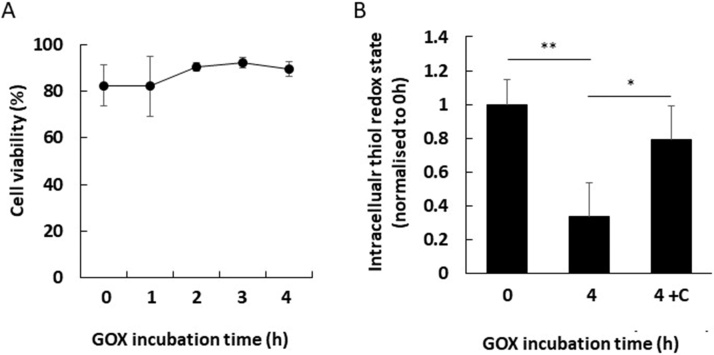
**The evaluation of exogenous GOX-derived H_2_O_2_ on Jurkat T-cell viability using trypan blue staining**. (A) Plot illustrates the mean Jurkat viability (%) following incubation with 5 mU/ml GOX at 0 h–4 h. To confirm that GOX-derived H_2_O_2_ was mediating the loss in reduced intracellular thiol, similar experiments were performed in the presence of 100 μg/ml catalase. (B) Plot illustrates the normalised mean loss of reduced intracellular thiol at 0 h compared with 4 h (GOX treated) and 4 h + C (GOX with catalase treated). Data illustrate that in the presence of catalase, intracellular thiol remains reduced. Data shown are mean +/− SD of the mean (n = 3). Data analysed by Kruskal–Wallis one-way analysis of variance with Dunn’s post-hoc test, *p < 0.05, **p < 0.01 and ***p < 0.001.
21 To demonstrate that the GOX generated H_2_O_2_ is mediating the loss in *reduced* thiols, the effect of catalase in ‘4 h’ vs ‘4 h + C’ treated cells can be observed on normalised data as shown in Fig. 2B. The data suggest that H_2_O_2_ mediates the loss in *reduced* intracellular thiols as measured by a loss in F5M (FL1) fluorescent signal.

Raw flow cytometric data are normalised to T0 (untreated). ^#^The FL1 MFI is for each time is divided against the FL1 MFI at 0 h. Data analysed by Kruskal–Wallis one-way analysis of variance with Dunn’s post-hoc test. Values compared with 0 h.
  Note: normalisation is performed by dividing the F5M signal by the F5M signal of non-GOX treated controls i.e. 0 h.
20 To confirm that GOX treatment is not inducing cell death, viable cell counts should be performed on GOX treated cells before or after flow cytometry acquisition as shown in Fig. 2A.

#### Method versatility and confocal microscopy confirmation that the F5M signal is intracellular

**Fig. 3:**
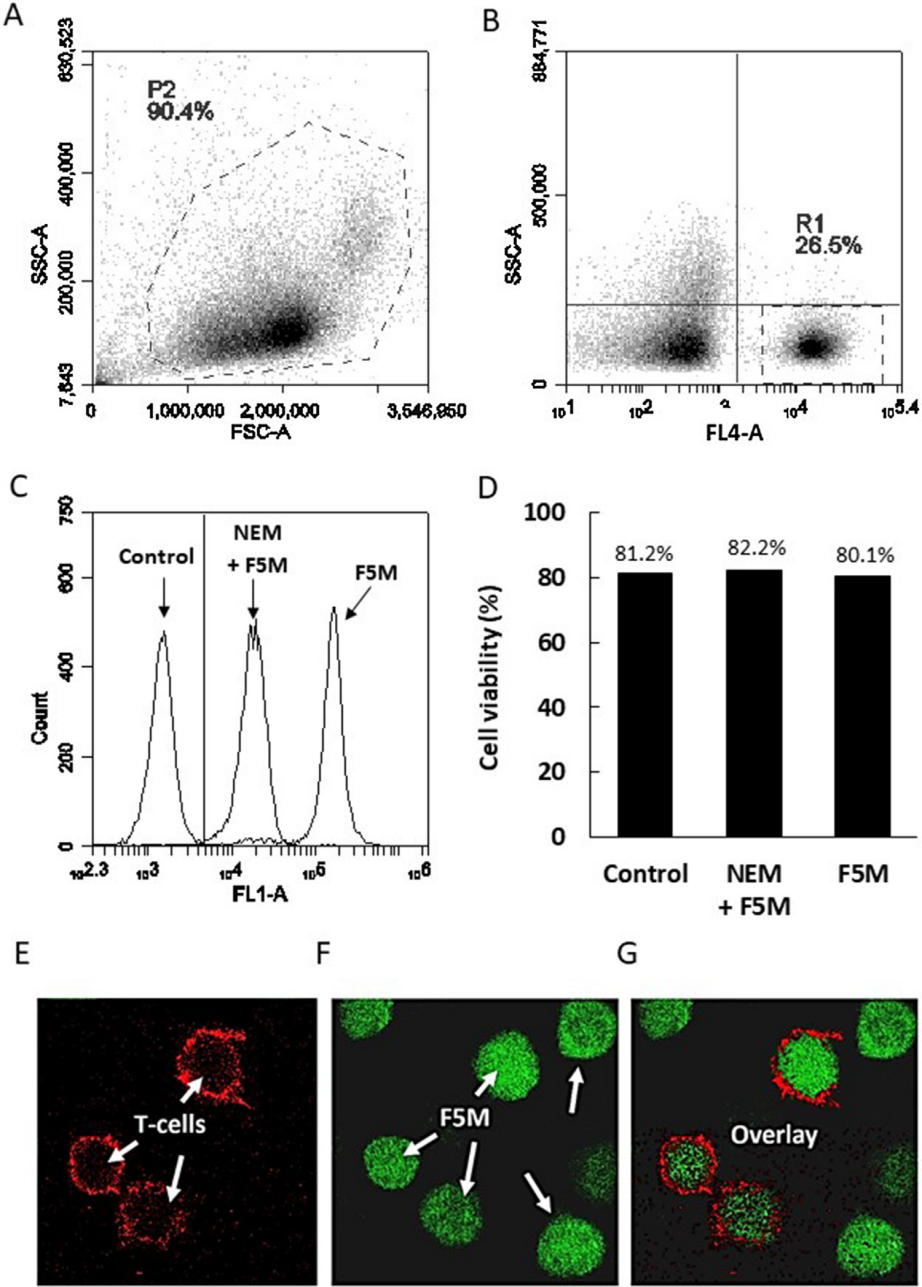
**Flow cytometric analysis of intracellular thiol redox status in primary human T-cells**. PBMC’s were prepared from venous blood using Ficoll-Paque density centrifugation and washed in d-PBS. Washed PBMC’s (10^6^ cells in 100 μl) were labelled with anti-human CD3-APC. PBMC’s were then treated with either 1 μM NEM followed by 0.1 μM F5M, or 0.1 μM F5M alone and analysed by flow cytometry. (A) Representative forward light scatter (FSC) vs side light scatter (SSC) flow cytometric profile with the treated PBMC’s identified in P2. (B) Gated on P2, CD3^+^ T-cells are shown in the FL4 parameter (laser: λ640 nm, filter: λ675/25 nm) in R1 (C). Panel shows representative fluorescence histograms depicting F5M fluorescence in control and treated T-cells (gated on R1). (D) T-cell viability was monitored before and after NEM and F5M treatment using trypan blue staining. Confocal microscopy was used to confirm (E) F5M cellular uptake in PBMCs, (F) surface staining for T-cells with anti-human CD3-APC and (G) F5M and T-cell overlay.
30 Next acquire the ‘NEM’ cells and create an FL1 (laser: λ488 nm, filter: λ530/30 nm) histogram plot gated on the T-cell population as seen in Fig. 3B. The peak shown on the FL1 histogram is representative of the F5M background staining and can be seen in Fig. 3C.
31 Finally acquire the ‘F5M’ cells. As shown in Fig. 3C, these cells fluoresce at an order of magnitude greater than ‘NEM’ cells.
32 To confirm viability, perform cell counts either before or after flow cytometry acquisition as described in steps 3 and 4. Fig. 3D shows little loss in cell viability post F5M treatment.
33 As shown in Fig. 3E–G, confocal microscopy is used to confirm that the F5M is entering the T-cells and therefore that the flow cytometric FL1 signal generated is representative of intracellular thiol redox status. This supports the notion that the extracellular environment is more ‘oxidising’, thus cysteine residues here will be to a great extent oxidised i.e. to disulphide or higher oxidation states. To visualise F5M labelled CD3^+^ T-cells by confocal microscopy, carefully affix a glass microscope slide to the microscope and pipette 50–100 μl of the final stained cell suspension as a droplet onto the upper surface. To limit evaporation gently place a thin coverslip (19 mm) on top of the droplet. Focus cells using a 63× oil immersion objective. Use both the argon (F5M) and helium-neon (CD3-APC) lasers at a previously determined appropriate power setting, and adjust the offset, gain, zoom and Z-position as necessary for optimal image quality. If available, opt for sequential scanning modes to allow simultaneous visualisation of both signals with minimal crosstalk between channels. DNA stains such as DAPI can be added to the staining protocol to identify nuclear regions where the appropriate excitation lines and channels are available.

To illustrate the versatility of our flow cytometric method, we provide a protocol for evaluating intracellular thiol oxidation in heterogeneous cell populations i.e. T-cells within a peripheral blood mononuclear cell (PBMC) fraction. Here we couple the cell surface staining of CD3 (T-cell marker) with F5M. Confocal microscopy was used to verify that F5M signal detected by flow cytometry was intracellular and not due to F5M binding at the plasma membrane. Venous blood from healthy donors following informed consent was collected by a trained phlebotomist into a 6 ml lavender stopper K2EDTA Vacationer^®^ tube (BD). All procedures from this point should be performed inside a Class 2 biological safety cabinet:

1 Transfer 6 ml venous blood to a fresh sterile universal tube and add an equal volume of Hank’s balanced salt solution (HBS); 12 ml total volume.
2 Add 3 ml Ficoll-Paque PLUS to three sterile 15 ml centrifuge tubes and carefully layer 4 ml of the diluted venous blood on top of the Ficoll-Paque PLUS.
3 Transfer the tubes to a bench-top centrifuge with swing-out rotor and centrifuge at 400 x g for 40 min at room temperature (18−20 °C) with the brake off.
4 Carefully remove the tubes from the centrifuge and return to the biological safety cabinet. The PBMC layer (white and delicate) will be visible at the Ficoll-Paque PLUS interface. Carefully remove the upper layer (plasma) to waste with a fresh sterile Pasteur pipette without disturbing the PBMC layer. Then using a fresh sterile Pasteur pipette, transfer the PBMC layer to a fresh 15 ml centrifuge tube. For each tube, remove the entire PBMC interface with as little of the Ficoll-Paque PLUS layer as possible.
5 Add 8 ml HBS to each tube and centrifuge at 100–300 × *g* for 10 min at room temperature.
6 Using a fresh sterile pipette, remove the supernatant from each tube taking great care not to disturb the PBMC pellet and suspend in 1 ml HBS. The PBMC’s can be pooled at this stage. Add sufficient HBS to the pooled cells to make 10 ml.
7 Centrifuge pooled PBMC’s at 100–300 × *g* for 10 min at room temperature.
8 Remove the supernatant taking great care not to disturb the PBMC pellet and suspend the pellet in 2 ml pre-warmed (37 °C) complete IMDM. Transfer the cells to an incubator set at 37 °C and 5% CO_2_ for subsequent analysis.
9 Prepare the F5M working solution for flow cytometric use in advance. Protect from light and store at 4 °C (up to 1 month) or at −20 °C for up to 1 year.
10 Remove the PBMC’s from the CO_2_ incubator and centrifuge at 100–300 × *g* in a benchtop refrigerated centrifuge with swing-out rotor for 5 min at room temperature.
11 Remove supernatant and suspend the pellet in 10 ml d-PBS (equilibrated to room temperature) and centrifuge at 100–300 × *g* for 10 min.
12 Remove supernatant and suspend the pellet in 1 ml FSB. Prepare a 1 in 20 dilution of the PBMC suspension for cell viability counting (5 μl PBMC in 95 μl FSB is sufficient) and transfer the remaining suspension to ice.
13 Mix 10 μl of the 1 in 20 PBMC dilution with 10 μl trypan blue solution and count viable cells using a haemocytometer.
14 Adjust the PBMC suspension to a density of 10^7^ viable cells/ml with pre-chilled (4 °C) FSB and add 100 μl (10^6^ cells) to three fresh 1.5 ml microcentrifuge tubes and centrifuge for 5 min at 100–300 × *g* and 4 °C.
  Note: At this point it is preferable to pellet cells using a microcentrifuge.
15 Remove supernatants and suspend each pellet in FSB to a final volume of 90 μl.
16 Add 10 μl of mouse anti-human CD3 (clone SK7) APC conjugate to two tubes and 10 μl of mouse IgG1 APC conjugate isotype control to the other. This step will surface stain the T-cells allowing intracellular vs extracellular differentiation during confocal microscopy.
17 Vortex all tubes briefly (1 to 2 s) and incubate on ice for 30 min protected from the light.
18 Add 1 ml d-PBS (chilled to 4 °C) to each tube and centrifuge for 5 min at 100–300 × *g* and 4 °C. Remove supernatants and then repeat this step.
19 Remove supernatants and add 1 ml d-PBS to the isotype control tube and 1 ml d-PBS to one of the anti-human CD3 tubes and label it ‘F5M’.
20 Add 1 ml of 1 μM NEM solution to the remaining anti-human CD3 tube. These cells will be used to set the background for the F5M T-cell stain. Label this tube ‘NEM’.
  Note: This concentration of NEM is based on 99+% purity. A lower grade can be used, which may require titrating to achieve the optimal concentration.
21 Incubate all tubes for 20 min on ice, protected from the light.
22 Centrifuge for 5 min at 100–300 × *g* and 4 °C.
23 Remove supernatants and suspend each pellet in 1 ml d-PBS and centrifuge for 5 min at 100–300 × *g* and 4 °C.
24 Remove supernatants and add 1 ml chilled d-PBS to the isotype control tube. These cells will be used to set the negative T-cell gate. Add 1 ml 0.1 μM F5M to both ‘NEM’ and ‘F5M’ tubes.
25 Incubate all three tubes for 20 min on ice, protected from the light.
26 Centrifuge for 5 min at 100–300 × *g* and 4 °C.
27 Remove supernatants and suspend each pellet in 1 ml pre-chilled FSB and centrifuge for 5 min at 100–300 × *g* and 4 °C. Repeat this step.
28 Finally remove supernatants and suspend each pellet in 100 μl pre-chilled FSB, keep on ice, protect from light and go to flow cytometer.
29 At the flow cytometer, acquire the ‘Ig-control’ cells first. These cells can be used to set the positive T-cell gate, which will appear in the FL4 parameter (laser: λ640 nm, filter: λ675/25 nm). Fig. 3A and B illustrates the gating strategy.

#### Analysing F5M labelled proteins extracted from viable cells

**Fig. 4:**
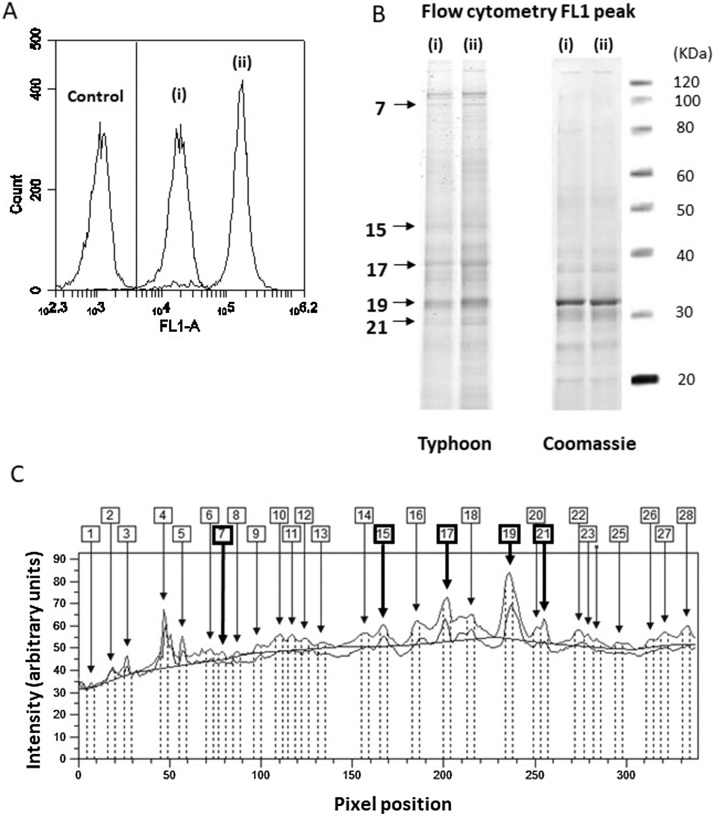
**Analysis of thiol containing redox active intracellular protein targets following NEM and F5M treatment of viable Jurkat T-cells**. (A) Panel shows a representative overlaid FL1 (laser: λ488 nm, filter: λ530/30 nm) histogram of Jurkat cells treated with 1 μM NEM + 0.1 μM F5M (i) and 0.1 μM F5M alone (ii). Respective protein extracts were prepared and separated by SDS-PAGE (protected from the light). Fluorescent protein bands were imaged using a Typhoon-9400 variable mode imager set at λ490–495 nm for excitation and λ515–520 nm for emission. The SDS-PAGE gel was counterstained with G-250 colloidal coomassie blue to confirm equal gel well loading. (B) Representative gel images for proteins extracted from flow cytometry FL1 peaks (i) and (ii). Typhoon images are presented along with respective Coomassie counterstained images. (C) Representative pixel peak profiles comparing fluorescent protein band intensity between (i) and (ii), lower and upper profiles respectively. Five corresponding gel bands (pixel peaks) are highlighted that differ in fluorescence intensity (no difference was observed for the respective coomassie counterstain).
2 Centrifuge the NEM and F5M treated Jurkat cells at 100–300 × *g* for 5 min at 4 °C.
3 Remove supernatants and add 10 ml pre-chilled FSB and centrifuge at 100–300 × *g* for 10 min at 4 °C.
4 Remove supernatants and suspend both pellets in 400 μl pre-chilled (4 °C) PBS and transfer to fresh microcentrifuge tubes for cell lysis by sonication.
  Note: Keep cell lysates on ice and protected from light at all times. This will minimise protein degradation and protect F5M from photo-bleaching.
5 Centrifuge tubes at 12,000 × *g* for 15 min at 4 °C and harvest supernatants into fresh microcentrifuge tubes on ice and protect from light. Perform protein quantitation on both cell extracts (Bradford assay [16] or equivalent is appropriate).
6 Add 20 μg of each protein extract to non-reducing NuPAGE^®^ LDS SDS-PAGE sample loading buffer made up to a final volume of 20 μl and heat at 70 °C for 10 min, protected from light. Do not add reducing agent e.g. 2-mercaptoethanol. Allow samples to cool before loading the Novex^®^ 4–20% gradient pre-cast tris-glycine gel.
7 Assemble the SDS-PAGE gel tank with the gel and add 1 x NuPAGE^®^ MOPS SDS-PAGE running buffer to upper and lower buffer chambers.
8 Load samples along with molecular weight marker and electrophorese at 180 V for 60–80 min, or until the dye front has run off. It is important to protect the gel and SDS-PAGE gel tank from light during this procedure.
9 Remove gel to a small tray containing dH_2_O and scan immediately using a Typhoon-9400 variable mode imager (or equivalent) set at λ490–495 nm for excitation and λ515–520 nm for emission.
10 Remove gel from Typhoon directly into SDS-PAGE gel fix solution and incubate for 15 min at room temperature with gentle agitation.
11 Rinse gel in dH_2_O and add G-250 colloidal coomassie blue reagent. Incubate overnight (10–20 h) at room temperature with gentle agitation.
12 Remove G-250 colloidal coomassie blue reagent and de-stain with dH_2_O for 2 h at room temperature with gentle agitation. Change the dH_2_O every 20 min.
13 Scan gel using a standard flatbed SDS-PAGE gel scanner and analyse the data along with the respective Typhoon fluorescence image data using ImageQuant (v8.1) or equivalent high resolution software. Fig. 4B shows Typhoon and respective coomassie gel images of proteins extracted from the NEM + F5M treated (i) and F5M alone treated (ii) Jurkat cells. Fig. 4C illustrates pixel peak profiles for NEM + F5M treated (i) and F5M alone treated (ii) extracted proteins.
14 Since F5M irreversible modifies cysteine thiols, identification of redox active proteins is permitted via mass spectrometry based on incremental mass shifts relating to F5M binding. Table 2 shows various intracellular redox active proteins identified from F5M treated Jurkat cells using LC/MS analysis. For the F5M mass shift, the formula C_24_H_13_NO_7_ (+427.069 Da) was used, since this relates to the mass difference expected for a Cys-thiol or Cys-thiolate modification by F5M.

The data in the previous section demonstrated that treating viable cells with F5M results in the labelling of solvent accessible intracellular *reduced* cysteine thiols [11]. This will include low molecular weight thiols such as glutathione [12], and solvent accessible cysteine thiols present in the native conformation of redox active proteins [13]. It would be expected that if a *reduced* cysteine thiol is modified through oxidation, F5M labelling will not occur, since F5M preferentially reacts with *reduced* cysteine thiols via the ‘Michael addition’ [14]. Thus identifying individual proteins using proteomic platforms e.g. mass spectrometry will add greater understanding of how individual cysteine thiols can modulate biological processes. To demonstrate this principle, proteins from Jurkat cells exposed to GOX-mediated H_2_O_2_ were separated by gel electrophoresis. Untreated vs. F5M SDS-PAGE separated proteins were then dissected from the gel for analysis by mass spectrometry. This protocol has potential to be adapted for use with next generation flow cytometers e.g. CyTOF [6,15]. The details of the experiment used are below:

1 Set up two treatments in 15 ml centrifuge tubes; (i) 2 × 10^6^ Jurkat cells with 1 μM NEM for 20 min followed by 20 μM F5M for 20 min, (ii) 2 × 10^6^ Jurkat cells with F5M only for 20 min. Carry out all F5M incubations as described in the above sections. Fig. 4A illustrates the level of F5M fluorescence for these treatments compared to untreated controls as analysed by flow cytometry.

**Table 2:** F5M labelled LC/MS identified intracellular redox active proteins.

Protein	Peptide Sequence Modified	Accession	Evidence of Involvement in Redox Regulation
Ser/Thr protein phosphatase 2A	ENVI**m**SQILP**c**IK – M5(oxidation) and C11(F5M addition)	B4DQY1	[17]
*N*-Methyl-d-aspartate subunit 2D	H**c**ASLELLPPPR – C2(F5M addition)	Q59G17	[18]
Human Leukocyte Antigen-DRB1	LPGGS**c**MAALTVTLMVLSSPLALA – C6(F5M addition)	O19734	[19]

Identification of key peptides from mass spectrometry analysis (LC/MC) of F5M labelled Cys that were present in Jurkat cells. Gel slices from proteins extracted from F5M treated cells were prepared following fluorescence SDS-PAGE analysis.
  Note: LC/MS analysis was outsourced to the Bristol Proteomics Facility (University of Bristol, UK).
